# Spatial Statistics for Understanding Tissue Organization

**DOI:** 10.3389/fphys.2022.832417

**Published:** 2022-01-28

**Authors:** Andrea Behanova, Anna Klemm, Carolina Wählby

**Affiliations:** Department of Information Technology and SciLifeLab, Centre for Image Analysis, Uppsala University, Uppsala, Sweden

**Keywords:** transcriptomics, spatial statistics, gene expression, tissue analysis, tissue organization, niches

## Abstract

Interpreting tissue architecture plays an important role in gaining a better understanding of healthy tissue development and disease. Novel molecular detection and imaging techniques make it possible to locate many different types of objects, such as cells and/or mRNAs, and map their location across the tissue space. In this review, we present several methods that provide quantification and statistical verification of observed patterns in the tissue architecture. We categorize these methods into three main groups: Spatial statistics on a single type of object, two types of objects, and multiple types of objects. We discuss the methods in relation to four hypotheses regarding the methods' capability to distinguish random and non-random distributions of objects across a tissue sample, and present a number of openly available tools where these methods are provided. We also discuss other spatial statistics methods compatible with other types of input data.

## 1. Introduction

A range of new imaging-based methods make it possible to explore the architecture of tissue samples both at the transcriptomics and proteomics level. Multiplexed *in situ* RNA detection methods (Ke et al., 2013; Shah et al., 2016; Codeluppi et al., 2018; Moffitt et al., 2018; Wang et al., 2018; Eng et al., 2019) map mRNA molecules at sub-cellular resolution, and multiplex immunohistochemical staining (Parra et al., 2019), make it possible to detect and identify a large number of different cell types in the same tissue sample, enabling the discovery of their functional role inside the tissue architecture (Grün and van Oudenaarden, 2015; Svensson et al., 2018). The first step toward further interpretation of the data is detection and decoding, or classification, of each individual object; in this case resulting in maps of the locations of either specific mRNA molecules or cells.

One of the key challenges in fully exploiting this type of spatially resolved data is the availability of appropriate computational methods. The second step in interpretation is to be able to quantify relationships and patterns in an unbiased and reproducible way, and provide confidence measures for observed patterns as compared to a more randomized organization. This is often referred to as spatial statistics.

In this mini-review, we focus on spatial statistics applicable to tissue data independent of image resolution. We start with the assumption that each observed object has a unique position in 2D tissue space, and is assigned a specific type (e.g., cell type or mRNA species). Further, we assume that we also want to take the tissue context, and distribution of other objects, into consideration.

**Figure 1:**
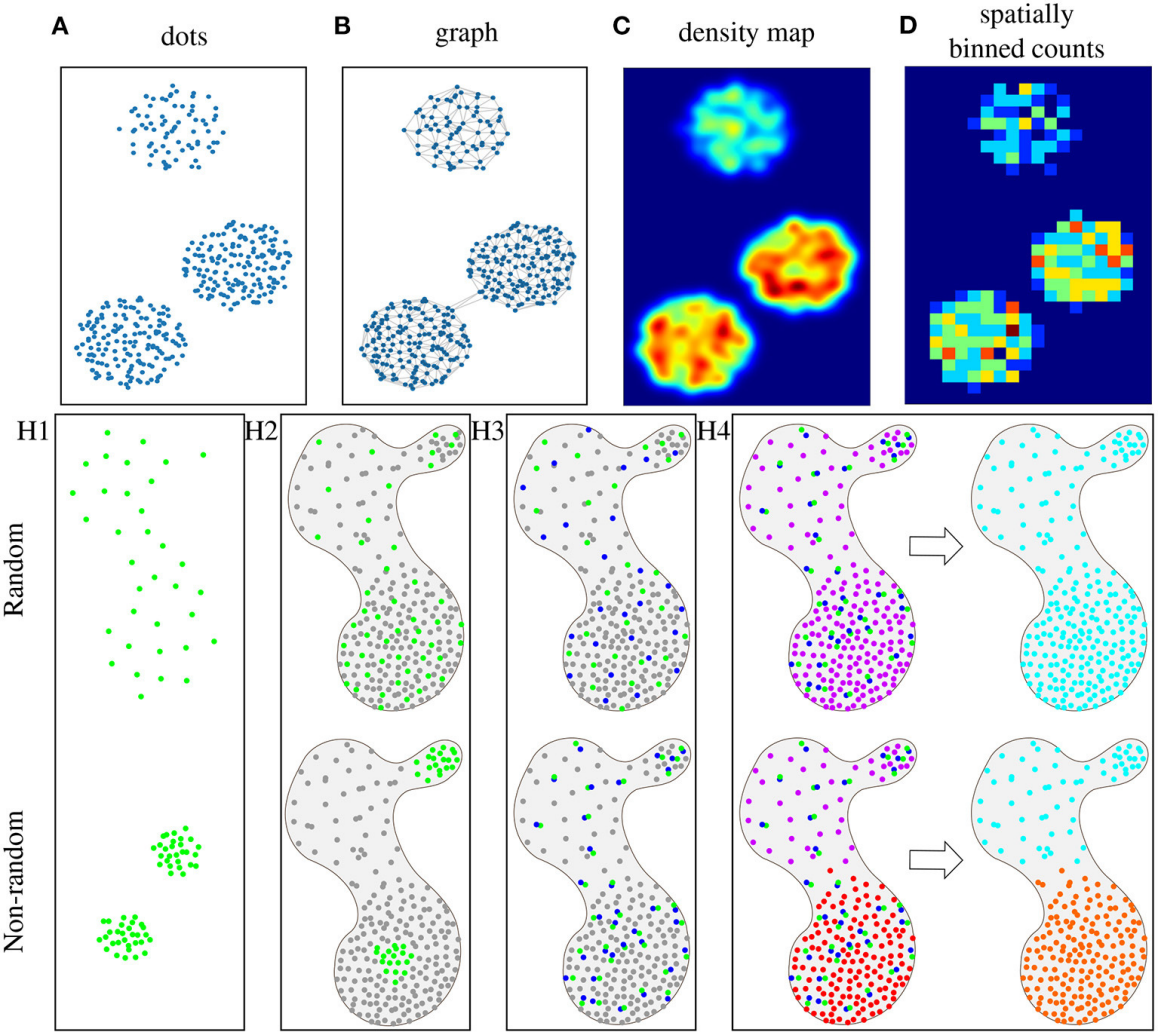
Schematic representations of objects, such as cells or mRNAs, in microscopy images, where each dot represents an object, and the color reflects the object type (where gray is an unspecified type). **(A)** Simple representation, where each dot has a specific location in 2D tissue space. **(B)** The same data represented as a graph, where each dot is a node, and nodes are connected based on a maximum distance criterion. **(C)** Dots can also be represented by a probability density map, where warmer colors represent more dense dots, or **(D)** as counts in fixed spatial bins. Here, bins are squares and warmer colors represent higher object counts per bin. Spatial statistics are used to prove four different hypothesis (with the top row representing the random case): (H1) Visualization of hypothesis H1: Objects of type A (green) are non-randomly distributed. (H2) Visualization of hypothesis H2: Objects of type A (green) are non-randomly distributed as compared to the distribution of other objects (gray) in the same tissue sample. (H3) Visualization of hypothesis H3: Objects of type A (green) and B (blue) are non-randomly distributed in relation to one another within the distribution of other objects (gray) in the same tissue sample. (H4) Visualization of hypothesis H4: There are groups of object types (multiple colors in “niches”) that are non-randomly distributed within the tissue sample.

Objects can then be presented either as dots, a graph, a density map, or spatially binned counts in tissue space, as illustrated in Figures 1A–D. In the dot representation (Figure 1A), a different color would typically be used for each species. In a graph representation (Figure 1B), neighboring objects are connected. These connections can be restricted to fulfill criteria, such as a maximum number of connections or distance, reflecting a hypothesis on a maximum distance for interaction. The density map representation (Figure 1C) translates the object distribution into a probability map, where high values represent high object concentrations, but the exact spatial location of objects is lost. Finally, different types of binning can be applied (Figure 1D), providing a lower-resolution map with counts of objects per bin.

We review methods that explore the null hypotheses of randomness for either a single type of objects, pairs of objects, or multiple types of objects. We have created a set of synthetic images describing different scenarios of object distributions within a tissue section, illustrating that the question of randomness is often relative. We first explore a single type of objects, as shown in Figure 1H1, and propose the hypothesis **H1**: Objects of type A are non-randomly distributed. In a biological context this could be, e.g., quantifying the distribution of immune cells in the presence or absence of an infection. If we take the tissue context (all objects of other types) into account, as shown in Figure 1H2, the hypothesis becomes **H2**: Objects of type A are non-randomly distributed as compared to the distribution of other objects in the same tissue sample. In a biological context this could be, e.g., distribution of a certain cell type in tumor and stroma areas of a tissue. Next, we consider two types of objects, and their potential interaction or repulsion. This is illustrated in Figure 1H3, and the hypothesis is **H3**: Objects of type A and B are non-randomly distributed in relation to one another within the distribution of other objects in the same tissue sample. In a biological context the question could, e.g., be whether cancer cells interact with endothelial cells or not. Finally, if there are multiple types of objects, we may want to see if certain groups of objects tend to coincide and form so-called 'niches' of unique combinations of objects in the tissue, as shown in Figure 1H4. In this case, we pose hypothesis **H4**: There are groups of object types (‘niches’) that are non-randomly distributed within the distribution of other objects in the tissue sample. This could be used for finding mRNAs that are co-expressed, where niches would then correspond to different cell types (Partel and Wählby, 2020).

In the following review, we group different spatial statistics methods according to what types of tissue patterns they investigate, and also summarize and discuss their theoretical ability to answer the four hypotheses we pose above.

## 2. Spatial Statistics on a Single Type of Object

In this section, we describe methods which are capable to test hypothesize H1 (non-random distribution) and H2 (non-random distribution, compared to other objects). The input data can be described as points in space determining the presence of an object. The main idea is to identify and characterize spatially variable objects.

### 2.1. Ripley's Function

Ripley's function (Ripley, 1976) measures whether objects with discrete positions in space (see Figure 1A) follow random, dispersed, or clustered patterns. For each object, the function counts how many other objects of the same type appear within a given distance. Subsequently, the object counts are averaged over the whole dataset and the number is compared with the number of objects one would expect to find based on a completely spatially random pattern (null hypothesis). If the average number of objects found within the given distance is greater than for a random distribution, the dataset is clustered (see green dots in Figure 1H1-down). If the number is smaller, the dataset is dispersed. Ripley's K function is generally calculated at multiple distances allowing detection of pattern distributions at multiple scales. For example, at short distances, the objects may be clustered, while at long distances, objects may be dispersed. This method can be used to test hypothesis H1 (non-random distribution).

### 2.2. Newman's Assortativity

Newman's assortativity (Newman, 2002) evaluates spatial organization using a graph (see Figure 1B) as input. The principle is to count existing connections between objects of the same category and compare these counts to the number of connections expected at random object distribution (null hypothesis). This method can be used to test hypothesis H1. Figure 1H1-up shows no significant difference in the number of connections compared to a random distribution. However, Figure 1H1-down indicates that there would be a significant difference in the number of connections than under the null hypothesis. The difference between Ripley's function and Newman's assortativity is that Ripley's forms an overall cluster analysis providing various evaluations using various distances while Newman's tests the dataset as one object determining clustered patterns. However, the graph structure in Newman's assortativity provides more flexibility since graph connections can be created by different techniques, such as k-nearest neighbors or Delaunay triangulation.

### 2.3. Centrality Scores

Centrality scores (Everett and Borgatti, 1999) are based on computational analysis to show object patterns in a graph representation (see Figure 1B). This provides awareness of complicated relations in large graphs. Figure 1H2 can be used as an example where green dots represent one object type (group members, e.g., immune cells) and gray dots represent members of all the other object types (non-group members, e.g., all types of tumor cells). This method can be used to test hypothesis H2. There are four different centrality scores: **Group degree centrality** is interpreted as a ratio of non-group members (gray) that are connected to group members (green). Higher values reveal random distribution. Lower values indicate more grouped objects. This measure helps to identify crucial clusters in a graph. **Group closeness centrality** computes how close the group (green) is to the non-group members (gray). It is defined as the amount of non-group members (gray) divided by the sum of all distances from the group (green) to all non-group members (gray). Higher values reveal random distribution. Lower values indicate more grouped objects. **Group betweenness centrality** calculates the quantity of shortest paths connecting two non-group members (gray) while passing through the group (green). This can be thought of as a measure of cell infiltration. **Average clustering coefficient** measures how likely the group members favor to cluster together.

## 3. Spatial Statistics on Two Types of Objects

In this section, we describe methods capable of testing hypothesis H3: objects of types A and B are non-randomly distributed in relation to one another within the distribution of other objects in the same tissue sample. The main idea is to identify if different types of objects are closer than what would be expected by chance. It is worth noting that physical closeness is no guarantee for interaction, but a non-random pattern may indicate involvement in similar processes.

### 3.1. Cluster Co-occurrence Ratio

Cluster co-occurrence ratio (Tosti et al., 2021) describes co-occurrence of two types of objects in the tissue. It measures the probability that an object of type *A* appears in a given distance from an object of type *B* by taking the ratio between occurrences of object type *A* within a distance from object type *B* and occurrences of object type *A* within a distance from object type *B* at random (null hypothesis). It is computed across multiple distances across the tissue area. It measures the probability that an object of type *A* appears in a given distance from conditioned object type *B*. Figure 1H3-up shows example of low cluster co-occurrence ratio and Figure 1H3-down shows example of high cluster co-occurrence ratio within a short distance.

### 3.2. Neighborhood Enrichment Test

Neighborhood enrichment test (Schapiro et al., 2017) identifies two non-randomly distributed objects types in relation to one another. The first step is to create a graph (see Figure 1B). Then two object types are selected (*A* and *B*) and the count of connections between *A* and *B* object types (*n*_*AB*_) is compared to random permutations of the objects (null hypothesis). The random configuration is set by keeping the object locations and reshuffling the object identities. Based on these estimates, expected means (μ_*AB*_) and standard deviations (σ_*AB*_) are calculated for each pair in the randomized dataset. Subsequently, a Z-score is calculated as, $Z_{AB}=\frac{n_{AB}-\mu_{AB}}{\sigma_{AB}}$. The Z-score indicates if an object type pair is over-represented (positive Z-score, see Figure 1H3-down) or over-depleted (negative Z-score, see Figure 1H3-up) in the connectivity graph. The difference between cluster co-occurrence ratio and neighborhood enrichment test is that cluster co-occurrence ratio evaluates various distances when determining if two objects types are in relation to one another while neighborhood enrichment test examines the dataset as one object determining object relation. However, the graph structure in Neighborhood enrichment test again provides flexibility since graph connections can be created by different techniques.

### 3.3. Object-Object Correlation Analysis

Object-Object Correlation Analysis (Stoltzfus et al., 2020) investigates the correlation of different object types within neighborhoods over the tissue. A neighborhood is a composition of objects inside a circular area. The neighborhoods' locations are uniformly allocated in a grid pattern throughout the space. The next step is to calculate the Pearson correlation coefficient of two types of objects within the neighborhoods. This method reveals which types of objects are associated with each other or unrelated to each other. Figure 1D shows an example of this neighborhood representation. The idea is to create this representation of two object types and then estimate the correlation coefficient across all overlapping neighborhoods.

## 4. Spatial Statistics on Multiple Types of Objects

In this section, we describe methods which are capable to test hypothesis H4 (existence of “niches”). The input data can be described as points in space determining the presence of the object types. The main idea is to identify if there are reoccurring spatial patterns, or 'niches' of objects, in the tissue.

### 4.1. Spatial Co-expression Patterns

Spatial co-expression patterns (Dries et al., 2021) identify robust patterns of object types that follow correlated spatial expression arrangements throughout the tissue. The first step is to smooth the object expression over the space by averaging in a grid or k-nearest neighbor technique. This results in a one density map for every object type as illustrated in Figure 1C. The next step is to calculate the Pearson correlation coefficient of the pair combinations of all object types (e.i., density maps). Subsequently, similarly co-expressed object types are clustered together into modules, and averaging them creates meta-object types to represent the similarly co-expressed object types.

### 4.2. Spage2vec

Spage2vec (Partel and Wählby, 2020) analyzes the spatial heterogeneity of complex patterns of objects. The input data is a graph (see Figure 1B), and it uses a graph representation learning technique based on a graph neural network (GNN). During training, the GNN learns the topological structure of each object's local neighborhood. It does not require labeled training data, but learns to find re-occurring patterns by comparing to a randomization of the data. After training, the observed patterns are summarized in a lower-dimensional embedding space that encapsulates high-dimensional information about each object's neighborhood. The last step is to cluster the multidimensional space using an unsupervised classification method (i.e., Leiden, Traag et al., 2019). Clusters represent combinations of object types that can be identified as specific domain types or ‘niches’. Figure 1H4-down shows an example, where different neighborhood compositions were identified as different niches. The types of discovered niches can be further identified by correlation between the object composition of the niches and e.g., in the case of *in situ* sequencing data an external dataset of scRNA-seq signatures. The approach has also been applied to detect niches in multiplex fluorescence microscopy data of tissue micro arrays (Solorzano et al., 2021).

### 4.3. Spot-Based Spatial Cell-Type Analysis by Multidimensional mRNA Density Estimation (SSAM)

SSAM (Park et al., 2021) was defined to identify tissue niches in transcriptomics data. The first step is to create probability maps of the object types. Kernel Density Estimation (KDE) with a Gaussian kernel is applied to every object type resulting in a density map for each object type (see Figure 1C). Then all the images are put into a stack creating a multi-channel image where each pixel is a vector describing the local expression profile. Next, group type signatures are computed by clustering using Louvain (Blondel et al., 2008) or DBSCAN (Ester et al., 1996), and outliers (vectors far from their cluster medoid) are removed. The cluster centroids represent the group-type signatures. The third step is to generate a group-type map. Each pixel in the vector field is classified according to the maximum correlation with the group-type signatures. The group-type signatures can be taken from the previous step or an external dataset, such as scRNA-seq. The fourth step is to identify the tissue niches with definite group-type composition. The composition is computed in a circular sliding window over the tissue and clustered by agglomerative hierarchical clustering, merging highly correlating clusters. Finally, each cluster represents a unique tissue niche, an example can be seen in Figure 1H4-down where two different niche types were found.

### 4.4. Vector Approach

Describing local neighborhoods as vectors of counts of object types has been suggested in several publications under multiple names (Stoltzfus et al., 2020; He et al., 2021; Salas et al., 2021). Here we refer to it as the vector approach. Its goal is to identify similar neighborhoods across the tissue sample. The first step is to define the neighborhoods. A neighborhood is a composition of object types inside a fixed area. The neighborhoods' locations can be uniformly allocated in a grid pattern throughout the space, constructed around each object from the dataset (Stoltzfus et al., 2020), based on Density peak clustering (He et al., 2021), or be defined by previously segmented tissue structures (Salas et al., 2021). Next, each neighborhood is presented as a vector containing counts of object types normalized, for example, by dividing each object count by the sum of all counts in the neighborhood (local normalization) or by dividing each object count by the sum of all the counts in the sample (global normalization). The normalized vectors are projected to a multidimensional space followed by clustering to identify niches. Examples of supervised clustering methods are common methods such as k-means and hierarchical clustering, or more advanced methods such as Self-Organizing Maps (Kohonen, 1982), Gaussian Distribution Model, or DBSCAN (Ester et al., 1996). Other clustering possibilities are unsupervised approaches such as Leiden (Traag et al., 2019) or Louvain (Blondel et al., 2008).

## 5. Toolboxes

Several toolboxes simplifying spatial statistics are available. Squidpy (Palla et al., 2021) includes four methods from this review: Ripley's function, Centrality scores, Cluster co-occurrence ratio, and the Neighborhood enrichment test. The toolbox PySpacell (Rose et al., 2019) includes methods such as Ripley's function and Newman's assortativity. CytoMap (Stoltzfus et al., 2020) includes Ripley's function, Object-Object correlation analysis and the Vector approach. Giotto focuses mostly on the data consisting of coordinates and quantitative information on multiple measurements per location, but also includes techniques as such as the Neighborhood enrichment test and Spatial co-expression patterns. The recently published Matisse (Salas et al., 2021) includes the Neighborhood enrichment test and the Vector approach. The toolbox histoCAT (Schapiro et al., 2017) includes the Neighborhood enrichment test, and Clustermap (He et al., 2021) includes the Vector approach. Table 1 summarizes these toolboxes and lists the hypotheses that each of the methods is capable of testing.

**Table 1:** Overview of the methods' functionality.

**Object**	**Method**	**H1**	**H2**	**H3**	**H4**	**Toolbox**
Single	Ripley's function	yes	no	no	no	Squidpy, PySpacell, CytoMap
	Newman's assortativity	yes	no	no	no	PySpacell
	Centrality scores	no	yes	no	no	Squidpy
Two	Cluster co-occurrence ratio	no	no	yes	no	Squidpy
	Neighborhood enrichment test	no	no	yes	no	Giotto, Matisse, Squidpy, histoCAT
	Object-Object Correlation Analysis	no	no	yes	no	CytoMap
Multiple	Spatial co-expression patterns	no	no	yes	yes	Giotto
	Spage2vec	no	no	no	yes	Spage2vec
	SSAM	no	no	no	yes	SSAM
	Vector approach	no	no	no	yes	CytoMap, ClusterMap, Matisse

## 6. Discussion

There are many published methods for spatial statistics. However, they differ in the type of input data they can handle. In this review, we focused on methods where the input data can be described as points in 2D tissue space representing the presence of different object types. Another type of input data consists of coordinates and quantitative information on multiple measurements per location, as in e.g., spatial transcriptomics (Larsson et al., 2021). Spatial statistics for exploring this type of data can focus on a single type of objects, with methods such as Binary Spatial extracts (BinSpect, Dries et al., 2021), Getis-Ord General G (Getis and Ord, 2010), Spatial pattern recognition via kernels (SPARK, Sun et al., 2020), spatialDE (Svensson et al., 2018), Trendsceek (Edsgärd et al., 2018), Geary's c (Geary, 1954) or Moran's I (Moran, 1950). In the case of more than a single type of object, there are other methods, such as Spatially informed ligand-receptor pairing (Dries et al., 2021), Object-Object Correlation Analysis (Stoltzfus et al., 2020) and Spatial domain detection (Dries et al., 2021) that can be applied for exploring co-locations, potential interactions and niche discovery.

The methods mentioned above are also applicable on the type of data we present in this paper (input data as points in space determining the presence of the object types) but the data would have to be pre-processed by transferring dots into spatially binned counts for all object types, as exemplified for a single object type in Figure 1D. With such a representation, spatial resolution would be lost, but data could be analyzed by methods such as Trendsceek and SPARK.

Many of the methods for analyzing multiple object types include clustering as a final step of the analysis. Different clustering algorithms might lead to different results when applied to the same data, and should be carefully selected. It should also be noted, that proving or disproving a hypothesis regarding spatial statistics will depend on quality and amount of input data. One should also keep in mind that a 2D section may not always be a good representation of a true 3D structure such as an organ.

## Author Contributions

AB, AK, and CW: conceptualization and investigation. AB and CW: writing. All authors have read and agreed to the published version of the manuscript.

## Funding

This research was funded by the European Research Council via ERC Consolidator grant CoG 682810 to CW and the SciLifeLab Bioimage Informatics Facility.

## Conflict of Interest

The authors declare that the research was conducted in the absence of any commercial or financial relationships that could be construed as a potential conflict of interest.

## Publisher's Note

All claims expressed in this article are solely those of the authors and do not necessarily represent those of their affiliated organizations, or those of the publisher, the editors and the reviewers. Any product that may be evaluated in this article, or claim that may be made by its manufacturer, is not guaranteed or endorsed by the publisher.
